# Association of sonographic features and molecular subtypes in predicting breast cancer disease outcomes

**DOI:** 10.1002/cam4.3305

**Published:** 2020-07-13

**Authors:** Haoyu Wang, Jiejie Yao, Ying Zhu, Weiwei Zhan, Xiaosong Chen, Kunwei Shen

**Affiliations:** ^1^ Department of General Surgery Comprehensive Breast Health Center Ruijin Hospital Shanghai Jiao Tong University School of Medicine Shanghai China; ^2^ Department of Ultrasonography Ruijin Hospital Shanghai Jiao Tong University School of Medicine Shanghai China

**Keywords:** breast cancer, molecular subtype, prognosis, ultrasonography, vertical orientation

## Abstract

**Background:**

Features in preoperative ultrasound could predict the prognosis of triple‐negative breast cancer (TNBC), while its prognostic value in other molecular subtypes of breast cancer (BC) was unknown. The study aimed to assess the prognostic value of preoperative sonographic features, including orientations, on long‐term outcomes in BC and its association with different molecular subtypes.

**Methods:**

Women diagnosed with invasive BC > 5 mm who underwent surgery were retrospectively reviewed. Clinical, pathological, and sonographic profiles were collected and recurrence‐free survival (RFS) and breast cancer‐specific survival (BCSS) were reported. Interactions between clinicopathological features and tumor orientations in predicting RFS and BCSS were analyzed. Competing risk model was performed to estimate prognostic values of sonographic features for RFS and BCSS.

**Results:**

A total of 2812 patients were included. With a median follow‐up of 60.0 months, 268 (9.5%) patients suffered from recurrences and 104 (3.7%) died of BC. The prognostic values of vertical orientation in predicting RFS (*P* = .001) and BCSS (*P* = .001) were strongly associated with molecular subtypes. Non‐TNBC tumors with vertical orientation had less recurrence events compared with parallel orientation (6.3% vs 8.7%, *P* = .035), whereas failed to predict disease outcomes in multivariate analysis (*P* > .05). Oppositely, in TNBC, vertical orientation was associated with worse RFS (HR = 3.50; 95% confidence interval [CI] 1.69‐7.24; *P* < .001) and BCSS (HR = 6.36; 95% CI 2.86‐14.14; *P* < .001) in multivariate analysis with a 5‐year RFS and BCSS of 73.4% and 74.6%. Meanwhile, vertical orientation was related with smaller tumor size (*P* < .001), human epidermal growth factor receptor 2 nonamplification (*P* < .001), and lower Ki‐67 expression (*P* = .001) among non‐TNBC population, whereas TNBC tumors with vertical orientation had a higher burden of axillary lymph node metastases (2.8 ± 1.0 vs 1.4 ± 0.2, *P* = .001).

**Conclusions:**

Prognostic values of sonographic orientation in predicting BC disease outcomes were associated with molecular subtypes. Vertical orientation in preoperative sonogram may serve as a prognostic biomarker for TNBC patients.

## Graphical abstract

Vertical orientation in preoperative ultrasound strongly interacted with molecular subtypes in predicting recurrence‐free survival (RFS) (*P* = .001) and breast cancer‐specific survival (BCSS) (*P* = .001). Vertical orientation independently related with worse RFS (HR = 3.50; 95% confidence interval [CI] 1.69‐7.24; *P* < .001) and BCSS (HR = 6.36; 95% CI 2.86‐14.14; *P* < .001), which associated with higher lymph node burden (2.8 ± 1.0 vs 1.4 ± 0.2, *P* = .001) for triple negative breast cancer (TNBC). Among non‐TNBC patients, vertical orientation was predictive for better RFS (5 year RFS 96.0% vs 92.9%, *P* = .035) which was related to smaller tumor size, human epidermal growth factor receptor 2 non‐amplification, and lower Ki‐67 expression (All *P* < .01).

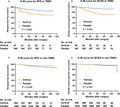

## 1 INTRODUCTION

Breast cancer (BC), as one of the major reasons of morbidity and mortality for women worldwide, is a heterogenous disease with variations in biological characteristics and clinical outcomes.^1^, ^2^ Researchers have introduced multiple prognostic biomarkers to predict recurrence risks and guide optimal treatment.^3^, ^4^, ^5^, ^6^, ^7^ Clinicopathological features including larger tumor size, presence of axillary lymph node (ALN) metastases, younger age, and higher histological grade have been proved to be associated with higher risk of recurrence.^3^, ^4^, ^5^ Classification of molecular subtypes based on the expression of estrogen receptor (ER), progesterone receptor (PR), human epidermal growth factor receptor 2 (HER2), and proliferation levels also helps predicting recurrence patterns and tailoring more personalized therapy for BC patients.^6^, ^7^ Aside from traditional clinicopathological traits, combined models of risk factors and gene panels have been established to further determine prognosis. For instance, Composite Risk model have been proved to predict long‐term outcomes for patients receiving adjuvant treatment, whereas the scoring system incorporating pretreatment clinical stage and post‐treatment pathologic stage as well as estrogen receptor status and tumor grade (CPS+EG system) showed predictive values in neoadjuvant settings.^8^, ^9^ Meanwhile, multiple gene arrays as 21‐gene recurrence score (RS) could identify patients of higher risk and optimize the choice of therapy.^10^ Furthermore, novel biomarkers have thrown fresh light on understanding biological behaviors of BC.^11^, ^12^ Expression level of tumor infiltrating lymphocytes and programmed death‐ligand 1 showed capability to predict prognosis^11^ and treatment response^12^ in BC, especially in triple‐negative breast cancer (TNBC). However, to further exploring the heterogeneous intrinsic profiles of BC, more biomarkers should be brought into practice.

Breast imaging may provide extra information of tumorous features for clinicians. Examinations including ultrasonography (US), mammography (MG), and magnetic resonance imaging (MRI), have been widely applied in screening and diagnosis of BC.^13^, ^14^ Compared with MG and MRI, advantages of US included well tolerance, no radiation, and cost‐effectiveness. What's more, US harbored higher accessibility in daily practice, which offered researchers more data resource to investigate. Studies have been done to evaluate the prognostic value of preoperative sonography features.^15^, ^16^, ^17^ It was reported that BCs classified as Breast Imaging Reporting and Data System (BI‐RADS) 4A category in screening US had higher risk of recurrence because tumors of higher proliferation may mimic the features of benign lesion.^15^ What's more, nonmass lesions with calcification^16^ have been previously reported to be associated with worse clinical outcomes. Notably, in our previous study, vertical orientation in preoperative ultrasound was found to be independently associated with inferior outcomes and higher ALN burden in TNBC patients.^17^ However, there was relatively limited data concerning whether sonographic features could predict disease outcomes in all subtypes of BC.

Thus, our study was to investigate the prognostic value of sonographic features in all subtypes of BC patients and further explore whether its prognostic value was associated with different molecular subtypes of BC.

## 2 MATERIALS AND METHODS

### 2.1 Patient cohort

Consecutive patients who received surgery and systematic adjuvant treatment in the breast health center in our university between January 2009 and December 2015 were retrospectively reviewed. Patients diagnosed pathologically as invasive BC larger than 5mm with preoperative ultrasound record in the hospital were eligible for inclusion. Exclusion criteria included male BC, simultaneous bilateral BC, neoadjuvant treatment, history of breast surgery, history of other malignancy, and diffusive or occult lesions in ultrasound because of their interference in image morphology and disease outcomes. The protocol was conducted under the terms of the Declaration of Helsinki and has been reviewed and approved by the Ethical Committee Review Board of Institution.

### 2.2 Ultrasound imaging technique and analysis

Preoperative sonograms were conducted by two physicians in the Ultrasonography Department majored in breast imaging with more than 10 years of experience. Images were collected by MyLab60 (Esaote) or Philip HD15 (Philips) with probes of 5‐12 MHz and then stored in the system of Digital Imaging and Communications in Medicine. Features including tumor orientation, shape, margin status, calcification, posterior acoustic patterns, architectural distortion, changes in Cooper's ligament, and color Doppler flow imaging were assessed and reported under the norm of 5th American College of Radiology (ACR) BI‐RADS Atlas.^18^

### 2.3 Pathological evaluation

Breast tumors were removed by surgery and evaluated by the Pathology Department. Estrogen receptor and PR positivity were defined if there was at least 1% staining in tumor nuclei.^19^ Status of HER2 was determined according to the 2018 ASCO/CAP (American Society of Clinical Oncology/College of American Pathologists) guideline for HER2 testing.^20^ Ki67 expression was scored as the percentage of positive invasive tumor cells with any nuclear staining and recorded as mean percentage of positive cells. Invasive BC was classified into five subtypes, including Luminal A, Luminal B/HER2‐, Luminal B/HER2+, HER2‐enriched, and TNBC according to 2013 St. Gallen Consensus.^6^ In this study, Luminal A, Luminal B/HER2‐, Luminal B/HER2+, and HER2‐enriched subtypes were classified as non‐TNBC.

### 2.4 Data collection and follow‐up

Clinical characteristics, pathological data, and follow‐up information of study population was recorded and retrieved from the BC database of the university. Clinicopathological variables were collected as follows: patients' age, menstrual status, family history, co‐morbidity, surgery types of breast and axillary, tumor size, histopathological types, ALN involvement, histological grade, ER status, PR status, HER2 status, lymphovascular invasion, Ki‐67 index, and adjuvant treatments. In this study, histopathological types were classified as invasive ductal carcinoma (IDC) and non‐IDC; histological grade was categorized as I‐II vs III and Ki‐67 index as ≤14% vs >14%.

Patients' follow‐up was done by BC‐specialized nurses and two endpoints including recurrence‐free survival (RFS) and breast cancer‐specific survival (BCSS) were selected into analysis. RFS was defined as the interval between the date of surgery and the date of locoregional recurrence, distant relapse, or contralateral BCs. BCSS was identified as the time period from BC surgery to the occurrence of BC‐specific deaths.

### 2.5 Statistical analyses

Analysis was conducted by IBM SPSS Statistics 25.0 (Windows version). Baseline characteristics were shown as numbers and percentages for categorical variables and as means and standard deviations for continuous variables. Comparison of categorical variables among subgroups was analyzed by Pearson's Chi‐square test (or Fisher's exact test when necessary), whereas continuous variables by independent sample *t* test. Stratified Mantel‐Haenszel test was conducted between sonographic orientation and clinicopathological features for RFS and BCSS and interaction *P* value was recorded. To avoid the influence of competing risk in survival analysis, we conducted competing risk analysis with “cmprsk” package in R (Windows 3.6.3 version). Gray's test was performed for univariate analysis and *P* value was recorded. Sonographic variables with *P* < .1 in univariate analysis and clinicopathological factors with potential prognostic value were then taken into multivariate analysis with Fine‐Gray model. Multivariate results were presented as subdistribution hazard ratio with 95% confidence interval (CI) with corresponding *P* values. Kaplan‐Meier curves was plotted for sonographic features in predicting patients' outcomes. All the tests were two‐sided and *P* < .05 was considered as significantly important.

## 3 RESULTS

### 3.1 Demographics and prognosis in the whole population

**FIGURE 1:**
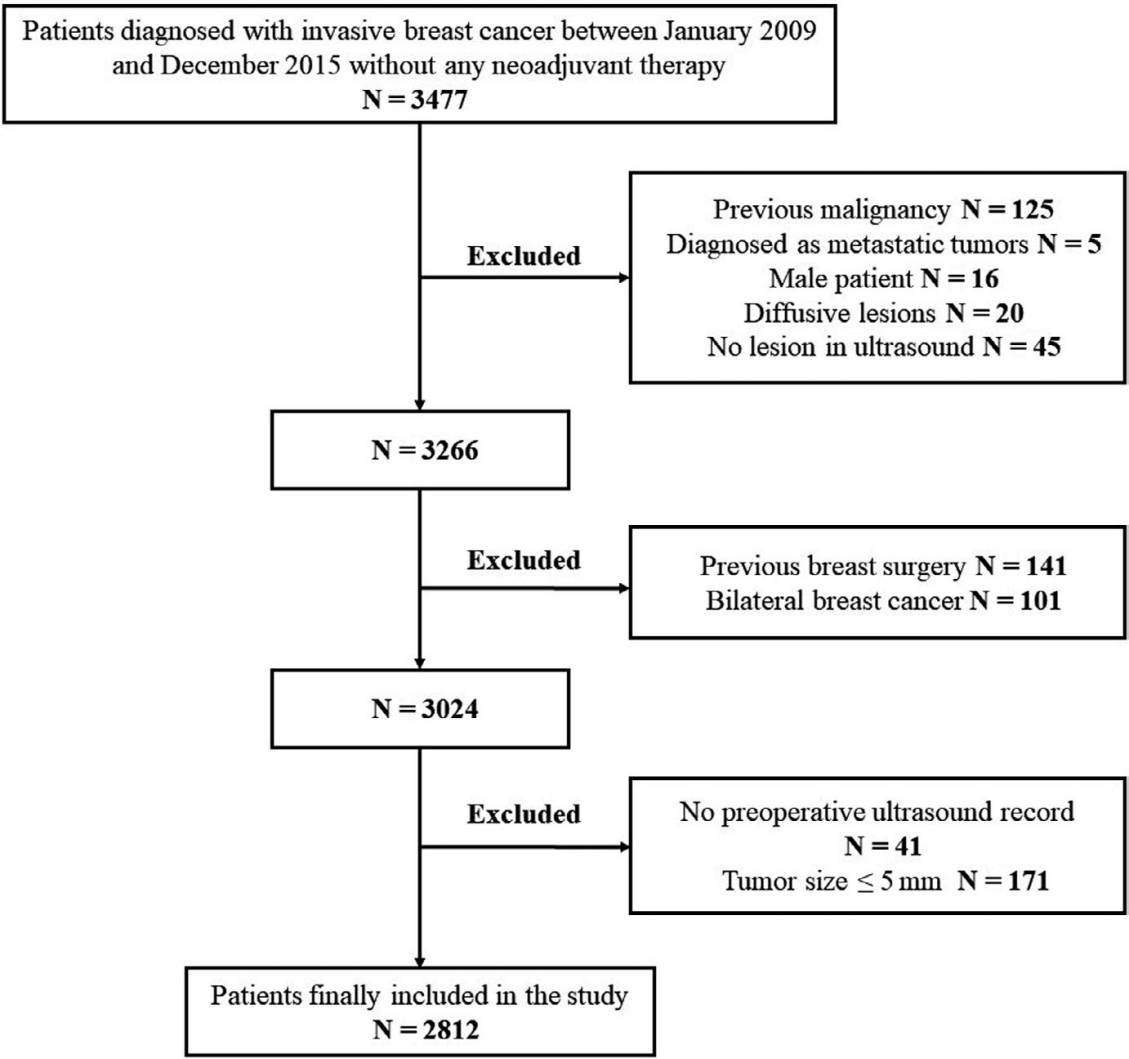
Flowchart of inclusion. Flowchart showed the inclusion and exclusion criteria of the study. In total, 2812 patients were included

A total of 3477 BC patients underwent surgery without neoadjuvant treatment, among which 2812 were finally included in the study (Figure 1). Characteristics of the patients and the tumors were listed in Table 1. The median age of the cohort was 55 years old (range 23‐93 years). One thousand five hundred sixty‐four (55.6%) of the patients had tumors ≤2.0 cm and 1002 (35.6%) had node positive diseases. There were 2481 (88.2%) patients had IDC and 1124 (40.0%) with grade III tumors. One thousand eight hundred ninety (67.2%) patients had hormone receptor‐positive diseases, whereas TNBC and HER2‐emplified BC were found in 416 (14.8%) and 293 (10.4%) cases, respectively. In total, 1988 (70.7%) patients underwent adjuvant chemotherapy, among which 1209 patients received regimens of anthracycline plus taxanes. The median time interval between preoperative sonography and breast surgery was 6 days and was similarly distributed among patients of different molecular subtypes and different sonographic orientations (*P* = .730).

**TABLE 1:** Clinical‐pathological features of the study population

Variables	Total	Non‐TNBC		*P* value
	N (%) (N = 2812)	Vertical (N = 270)	Parallel (N = 1913)	
Age (y)				0.003*
<35	91 (3.3)	1 (0.4)	68 (3.6)	
35‐44	428 (15.2)	29 (10.7)	308 (16.1)	
45‐54	802 (28.5)	74 (27.4)	545 (28.5)	
55‐64	821 (29.2)	93 (34.4)	564 (29.5)	
≥65	670 (23.8)	73 (27.0)	427 (22.3)	
Menstrual status				0.002*
Pre/perimenopausal	1085 (38.6)	81 (30.0)	765 (40.0)	
Postmenopausal	1727 (61.4)	189 (70.0)	1148 (60.0)	
Surgery time interval				0.730
≤6 d	1899 (67.5)	187 (69.3)	1305 (68.2)	
>6 d	913 (32.5)	83 (30.7)	608 (31.8)	
Histopathological type				0.549
IDC	2481 (88.2)	240 (88.9)	1676 (87.6)	
Non‐IDC	331 (11.8)	30 (11.1)	237 (12.4)	
Lymphovascular invasion				0.053
Absent	2669 (94.9)	263 (97.4)	1811 (94.7)	
Present	143 (5.1)	7 (2.6)	102 (5.3)	
Histological grade				0.007*
I	120 (4.2)	34 (13.5)	153 (9.0)	
II	1251 (44.2)	162 (64.5)	1035 (61.1)	
III	1124 (40.0)	55 (21.9)	506 (29.9)	
NA	317 (11.3)	19 (7.0)	219 (11.4)	
Tumor size				<0.001*
≤2 cm	1564 (55.6)	203 (75.2)	1031 (53.9)	
>2 cm	1248 (44.4)	67 (24.8)	882 (46.1)	
Lymph nodes involvement				0.142
Absent	1810 (64.4)	179 (66.3)	1179 (61.7)	
Present	1002 (35.6)	91 (33.7)	733 (38.3)	
Mean ± SE	1.7 ± 0.1	1.5 ± 0.2	1.7 ± 0.1	0.223
Ki‐67 (%)				<0.001*
≤14	987 (35.1)	140 (51.9)	709 (37.1)	
>14	1825 (64.9)	130 (48.1)	1203 (62.9)	
Mean ± SE	28.7 ± 0.5	20.0 ± 1.2	25.3 ± 0.5	0.005*
TNM stage				0.003*
I	1196 (42.6)	141 (52.2)	789 (41.3)	
II	1234 (43.9)	100 (37.0)	843 (44.1)	
III	382 (13.5)	29 (10.7)	280 (14.6)	
ER				0.001*
Negative	766 (27.2)	20 (7.4)	289 (15.1)	
Positive	2046 (72.8)	250 (92.6)	1623 (84.9)	
PR				<0.001*
Negative	1160 (41.3)	58 (21.5)	614 (32.1)	
Positive	1652 (58.7)	212 (78.5)	1298 (67.9)	
HER2				<0.001*
Negative	1996 (76.8)	229 (84.8)	1374 (71.9)	
Positive	603 (23.2)	41 (15.2)	538 (28.1)	
Chemotherapy				**<0.001** *
No	814 (28.9)	115 (42.6)	570 (29.8)	
Yes	1988 (70.7)	155 (57.4)	1337 (69.9)	
A plus T	1209 (43.0)	76 (28.2)	778 (40.7)	
A‐containing	176 (6.3)	17 (6.3)	123 (6.4)	
T‐containing	469 (16.6)	46 (17.0)	361 (18.9)	
Other regimens	134 (4.8)	16 (0.6)	75 (0.4)	
NA	10 (0.4)	0 (0.0)	6 (0.3)	

Note

Abbreviations: A, anthracycline; ER, estrogen receptor; HER2, human epidermal growth factor receptor2; IDC, invasive ductal carcinoma; LVI, lymphovascular invasion; NA, not applicable; PR, progesterone receptor; T, taxane.

*P* values in bold and asterisk meant significant difference.

With a median follow‐up time of 60.0 months, a total of 268 (9.5%) recurrences events and 104 (3.7%) BC‐specific deaths were reported in the whole population. The 5‐year rate of RFS and BCSS were 92.3% and 96.0%, respectively. Regarding RFS events, there were 46 (1.6%) local and regional recurrences, 193 (6.9%) distant metastases, and 29 (1.0%) contralateral BCs.

### 3.2 Interactions between clinicopathological features and sonographic orientations in predicting disease outcomes

**FIGURE 2:**
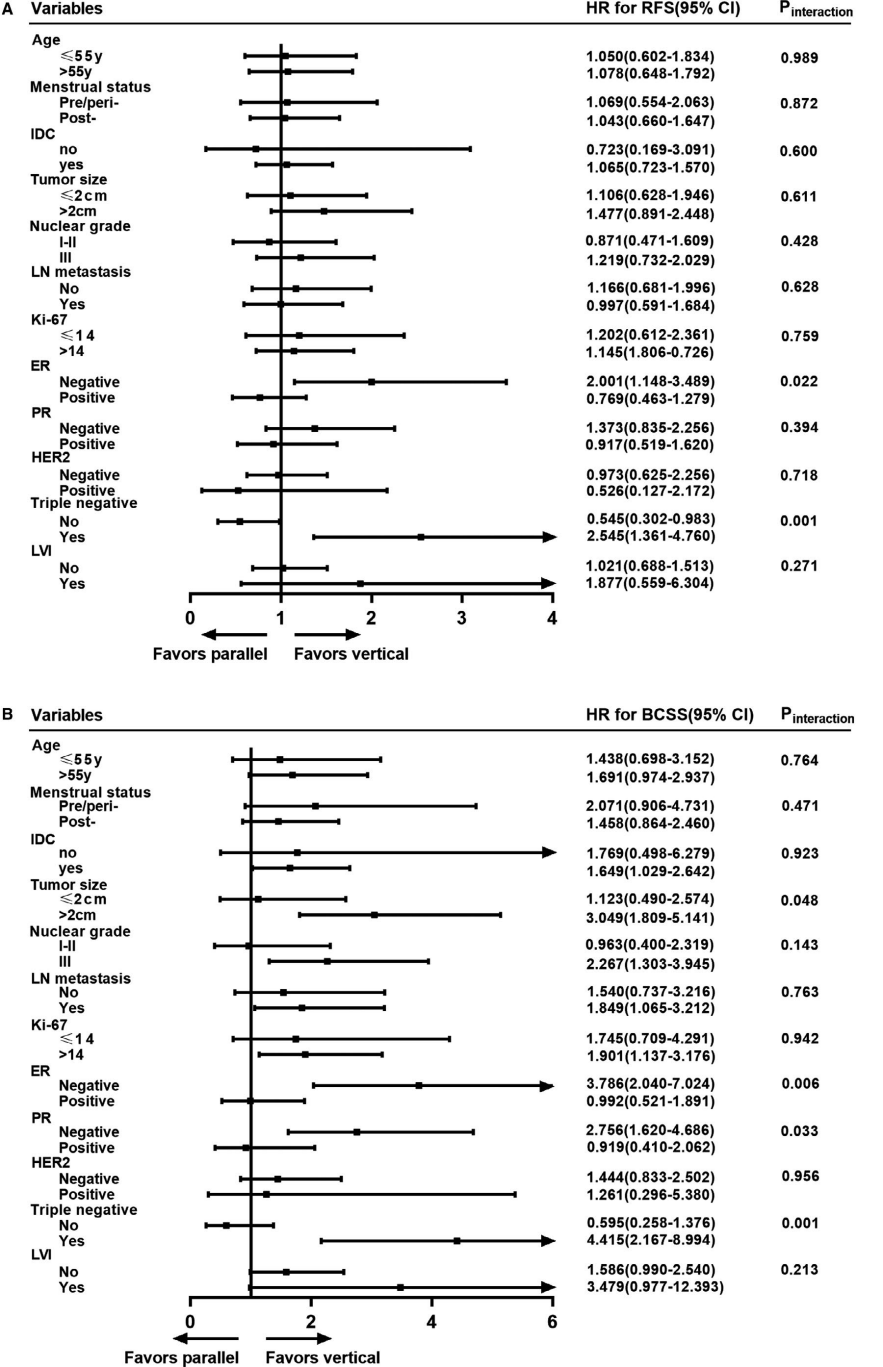
Interaction between sonographic orientation and clinical‐pathological features in predicting patient outcomes. Forest plots for interaction analysis between sonographic orientation and clinical‐pathological characteristics in predicting (A) RFS and (B) BCSS. The *P* value for interaction among each group was shown. The position of black squares represented the HR; the horizonal lines represented 95% CI. BCSS, breast cancer‐specific survival; CI, confidence interval; ER, estrogen receptor; HER‐2, human epidermal growth factor receptor 2; HR, hazard ratio; IDC, invasive ductal carcinoma; LN, lymph node; LVI, lymphovascular invasion; PR, progesterone receptor; RFS, recurrence‐free survival

Interactions between sonographic orientation and conventional clinicopathological features were explored to predict disease outcomes (Figure 2). No significant interactions were detected between clinicopathological covariates and sonographic orientations for neither RFS nor BCSS, including menopausal status (pre‐/peri‐ vs postmenopausal), histopathological types (IDC vs non‐IDC), ALN metastases (no vs yes), histological grade (I‐II vs III), and Ki‐67 percentage (≤14% vs >14%). Remarkably, there were significant interactions between vertical orientation and molecular subtypes (non‐TNBC vs TNBC, *P* = .001) and ER status (negative vs positive, *P* = .022) in predicting RFS (Figure 2A). The HR for RFS for vertical vs parallel orientation was 0.55 (95% CI 0.30‐0.98) in non‐TNBC subgroup and 2.55 (95% CI 1.36‐4.76) in TNBC subgroup. Similarly, for patients with ER‐negative BC, the HR for RFS for vertical vs parallel orientation was 2.00 (95% CI 1.15‐3.49).

Regarding BCSS, tumor size (≤2.0 cm vs >2.0 cm, *P* = .048), ER status (negative vs positive, *P* = .006), PR status (negative vs positive, *P* = .033), and molecular subtypes (non‐TNBC vs TNBC, *P* = .001) showed significant interactions with vertical orientation in predicting BC‐specific deaths (Figure 2B). Among patients with tumors larger than 2.0cm, vertical orientation predicted worse BCSS compared with parallel orientation (HR = 3.05, 95% CI 1.81‐5.14). Consistent with RFS, tumors with vertical orientation had inferior BCSS both in TNBC (HR = 4.42, 95% CI 2.17‐8.99) and ER‐negative BC (HR = 3.79, 95% CI 2.04‐7.02).

### 3.3 Sonographic features predict patients’ outcomes in different subsets of BC

**FIGURE 3:**
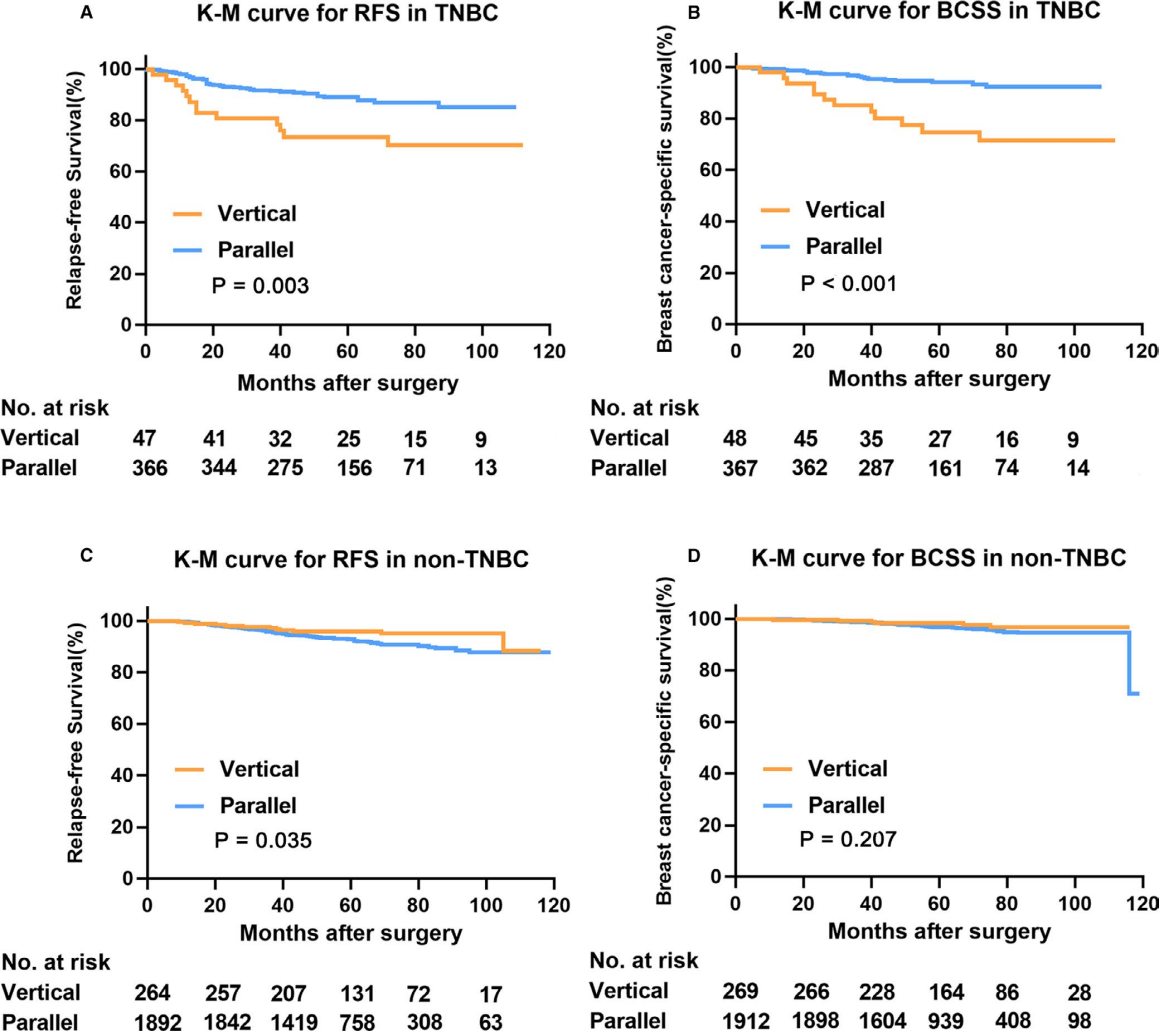
Kaplan‐Meier survival curves for sonographic orientation in TNBC and non‐TNBC patients. Kaplan‐Meier curves stratified by sonographic orientations were illustrated for TNBC and non‐TNBC patients, respectively. Vertical orientations showed worse (A) RFS (*P* = .003) and (B) BCSS (*P* < .001) in TNBC patients. Meanwhile, vertical orientation indicated (C) favorable RFS (*P* = .032) and (D) similar BCSS (*P* = .207) compared with parallel tumors in non‐TNBC patients. BCSS, breast cancer‐specific survival; K‐M, Kaplan‐Meier; RFS, recurrence‐free survival; TNBC, triple‐negative breast cancer

Univariate analyses of sonographic orientations and patient outcomes in total population and subgroups of BC were demonstrated in Table 2. Vertical orientation failed to predict RFS (*P* = .881), but was significantly associated with inferior BCSS (*P* = .032). When it came to subgroup analysis, in TNBC patients, vertical orientation was independently predictable for both worse RFS (*P* = .003) and BCSS (*P* < .001) with a 5‐year RFS and BCSS of 73.4% and 74.7%, respectively, which was worse than TNBC patients with parallel orientation feature (5‐year RFS 89.0%, BCSS 94.1%) (Figure 3). Similar results were also found in ER‐negative BC that vertical orientation was associated with unfavorable RFS (*P* = .015) and BCSS (*P* < .001) (Figure S1). On the other hand, in non‐TNBC patients, tumors with vertical orientation showed superior RFS compared with parallel orientation (5‐year RFS 96.0% vs 92.9%, *P* = .035), whereas orientations were not predictable for BCSS (*P* = .207) (Figure 3). Additionally, prognostic effects of sonographic orientation on RFS and BCSS were analyzed in Luminal‐A like, Luminal‐B like, and HER2‐enriched BC relatively. Vertical orientation failed to predict disease outcomes in those subtype of BC (All *P* > .05) (Table S3; Figure S2).

**TABLE 2:** Univariate analysis of orientation and patient outcomes in subgroups of BC patients

Endpoints	Vertical		Parallel		*P* value
	Events N	5 y rate %	Events N	5 y rate %	
RFS					
All population	40	91.5	228	92.4	0.881
TNBC	14	73.4	43	89.0	0.003*
Non‐TNBC	17	96.0	166	92.9	0.035*
ER‐negative	17	79.3	81	89.3	0.015*
ER‐positive	23	94.5	147	93.6	0.300
BCSS					
All population	25	94.0	96	96.3	0.032*
TNBC	12	74.7	22	94.1	<0.001*
Non‐TNBC	6	98.4	64	96.8	0.207
ER‐negative	14	80.7	37	94.7	<0.001*
ER‐positive	11	97.4	59	96.9	0.970

Note

Abbreviations: BCSS, breast cancer‐specific survival; ER, estrogen receptor; RFS, recurrence‐free survival; TNBC, triple‐negative breast cancer.

*P* values in bold and asterisk meant significant difference.

Besides tumor orientations, univariate analysis of other ultrasound features was also performed in total population and subgroups stratified by molecular subtypes (TNBC vs non‐TNBC) and ER status (negative vs positive). None of the sonographic features besides tumor orientations were associated with RFS and BCSS in TNBC (All *P* > .05) (Table 3). Although sonographic features cannot predict disease outcomes for TNBC patients, several features as irregular shape and BI‐RADS 5 category showed predictive value in other subgroups of BC. Irregular shapes were found to be associated with worse RFS both in non‐TNBC (*P* = .032) and ER‐positive BC (*P* = .022), whereas failed to predict BCSS in neither non‐TNBC (*P* = .235) nor ER‐positive BC (*P* = .142) (Table 3; Table S1). On the other hand, tumors with BI‐RADS 5 category showed unfavorable RFS (*P* < .001) and BCSS (*P* < .001) in total population, non‐TNBC, ER‐negative BC, or ER‐positive BC (All *P* < .01) (Table 3; Table S1).

**TABLE 3:** Univariate analysis of sonographic features and clinical outcomes in total population, non‐TNBC and TNBC patients

Variables	Total		Non‐TNBC		TNBC	
	RFS	BCSS	RFS	BCSS	RFS	BCSS
Orientation (vertical vs parallel)	0.881	0.032*	0.035*	0.207	0.003*	<0.001*
Shape (irregular vs regular)	0.020*	0.110	0.032*	0.235	0.695	0.569
Margin	0.114	0.375	0.071	0.644	0.866	0.940
Angular vs circumscribed	0.025*	0.090	0.070	0.224	0.491	0.725
Spiculate vs circumscribed	0.308	0.369	0.663	0.859	0.359	0.471
Micro‐lobulated vs circumscribed	**0.028**	0.148	0.075	0.250	0.354	0.562
Distinct vs circumscribed	0.295	0.547	0.435	0.462	0.401	0.889
Posterior acoustic pattern	0.494	0.834	0.154	0.819	0.487	0.954
Shadowing vs no change	0.194	0.494	0.136	0.466	0.860	0.627
Enhancement vs no change	0.692	0.852	0.176	0.956	0.167	0.922
Mixed change vs no change	0.268	0.604	0.061	0.615	0.627	0.857
Calcification (yes vs no)	0.345	0.382	0.290	0.531	0.677	0.362
Architectural distortion (yes vs no)	0.378	0.703	0.091	0.312	0.762	0.253
Change in Cooper's ligament (yes vs no)	0.419	0.678	0.117	0.242	0.606	0.274
CDFI	0.881	0.384	0.763	0.456	0.489	0.122
Low vs no	0.630	0.915	0.468	0.551	0.269	0.041*
High vs no	0.619	0.578	0.543	0.335	0.234	0.102
BI‐RADS (4B, 4C, 5 vs 4A)	0.080	0.134	0.197	0.414	0.372	0.441
BI‐RADS (5 vs 4A, 4B, 4C)	<0.001*	<0.001*	0.001*	0.001*	0.192	0.069

Note

Abbreviations: BCSS, breast cancer‐specific survival; BI‐RADS, breast imaging reporting and data system; CDFI, color Doppler flow imaging; RFS, recurrence‐free survival; TNBC, triple‐negative breast cancer.

*P* values in bold and asterisk meant significant difference.

Multivariate analyses were demonstrated both for sonographic and clinicopathological characteristics in predicting clinical outcomes. Regarding clinicopathological features, tumors >2.0 cm (HR = 1.76; 95% CI 1.27‐2.52; *P* < .001), ALN metastases (HR = 2.27; 95% CI 1.61‐3.19; *P* < .001), histological grade III (HR = 1.44; 95% CI 1.03‐2.00; *P* = .033), and PR negativity (HR = 1.78; 95% CI 1.28‐2.48; *P* < .001) were independently associated with worse RFS in non‐TNBC. Meanwhile, tumors >2.0 cm (HR = 1.84; 95% CI 1.08‐3.13; *P* = .026), ALN metastases (HR = 2.93; 95% CI 1.69‐5.06; *P* < .001), and PR negativity (HR = 2.02; 95% CI 1.21‐3.38; *P* = .007) also predicted unfavorable BCSS. However, after adjustment of these clinicopathological features, vertical orientation failed to predict neither RFS (HR = 0.69; 95% CI 0.37‐1.29; *P* = .240) nor BCSS (HR = 0.57; 95% CI 0.20‐1.62; *P* = .300) among non‐TNBC patients (Table 4). On the contrary, vertical orientation was independently associated with inferior RFS (HR = 3.50, 95% CI 1.79‐7.24; *P* < .001) and BCSS (HR = 6.36, 95% CI 2.86‐14.14; *P* < .001) in TNBC patients (Table S2). Additionally, other sonographic features including irregular shape and BI‐RADS 5 category failed to predict disease outcomes in multivariate analysis (Table 4; Table S2).

**TABLE 4:** Multivariate analysis of sonographic and clinicopathological features for RFS and BCSS in non‐TNBC patients

Categories	RFS		BCSS	
	SHR (95% CI)	*P* value	SHR (95% CI)	*P* value
Orientation		0.240		0.300
Parallel	1.00		1.00	
Vertical	0.69 (0.37‐1.29)		0.57 (0.20‐1.62)	
Tumor size		**<0.001** *		**0.026** *
≤2 cm	1.00		1.00	
>2 cm	1.79 (1.27‐2.52)		1.84 (1.08‐3.13)	
ALN metastases		**<0.001** *		**<0.001** *
No	1.00		1.00	
Yes	2.27 (1.61‐3.19)		2.93 (1.69‐5.06)	
Histological grade		**0.033** *		0.061
I‐II	1.00		1.00	
III	1.44 (1.03‐2.00)		1.63 (0.98‐2.71)	
PR		**<0.001** *		**0.007** *
Positive	1.00		1.00	
Negative	1.78 (1.28‐2.48)		2.02 (1.21‐3.38)	

Abbreviation: ALN, axillary lymph node; BCSS, breast cancer‐specific survival; CI, confidence interval; LVI, lymphatic vessel invasion; PR, progesterone receptor; RFS, recurrence‐free survival; SHR, subdistribution hazard ratio; TNBC, triple‐negative breast cancer.

*P* values in bold and asterisk meant significant difference.

### 3.4 Distribution of clinicopathological features in different ultrasound orientations

Distribution of clinicopathological features in tumors with different orientations were analyzed according to different molecular subtypes. In non‐TNBC patients, vertical orientation was significantly associated with smaller tumor size (*P* < .001), ER positivity (*P* < .001), PR positivity (*P* < .001), HER‐2 nonamplification (*P* < .001), and lower Ki‐67 expression (*P* = .005) (Table 1). Similarly, cases with tumor size ≤ 2.0 cm, HER‐2 nonamplification, and lower Ki‐67 expression were more likely to present as vertical orientation in ultrasound in ER‐positive BC (All *P* < .05) (Table S5). Regarding TNBC, vertical orientation was related with a higher burden of ALN metastases compared with parallel orientation (2.8 ± 1.0 vs 1.4 ± 0.2, *P* = .001) (Table S4), which was consistent with the result in ER‐negative BC (2.9 ± 0.2 vs 1.7 ± 0.1, *P* = .049) (Table S5).

## 4 DISCUSSION

In this study, the prognostic value of detailed sonographic features was explored and compared in all molecular subtypes of BC. It was found that the effects of tumor orientation in ultrasound in predicting RFS and BCSS were significantly associated with molecular subtypes. In TNBC patients, vertical orientation was independently associated with unfavorable RFS (HR = 3.50; 95% CI 1.69‐7.24; *P* < .001) and BCSS (HR = 6.36; 95% CI 2.86‐14.14; *P* < .001); oppositely, vertical orientation showed no prognostic value for non‐TNBC patients although it was strongly correlated with less proliferative pathological features. It was indicated that the prognostic value of sonographic orientation was associated with molecular subtypes and vertical orientation can be served as a prognostic biomarker for TNBC patients.

Efforts have been made in exploring the predictive and prognostic value of preoperative sonography features in BC patients while few consensuses have been reached by now. Kim SY et al reviewed 501 BC patients detected at screening ultrasound and demonstrated that tumors classified as BI‐RADS 4A category were associated with higher risk of recurrence compared with other categories (HR = 5.11, 95% CI 1.53‐17.20; *P* = .008).^15^ However, detailed features of ultrasound were not evaluated in this study. In our study, tumors with BI‐RADS 4A category failed to predict disease outcomes in terms of RFS (*P* = .080) and BCSS (*P* = .134). The conflicting results between two studies may be explained by the different proportion of BI‐RADS 4A tumors that 30% (129/425) of tumors in Kim's cohort were assessed as BI‐RADS 4A, whereas only 4.6% (128/2812) were 4A category in our study. Oppositely, compared with BI‐RADS 4 category (including 4A, 4B, and 4C), tumors with BI‐RADS 5 indicated unfavorable RFS (*P* < .001) and BCSS (*P* < .001) in univariate analysis. In terms of specific sonographic features, patterns of blood flow after treatment including peak systolic velocity, pulsatility index, and resistive index have been reported to be a surrogate predictor for treatment response and disease‐free survival in BC patients.^21^, ^22^ However, as one of the most important prognostic biomarkers, molecular subtypes were not put into analysis in these studies. Additionally, in a large cohort of 3112 patients from Korea, nonmass lesions with calcification at US were found to be independently related with inferior RFS (HR = 1.4, 95% CI 1.1‐1.8; *P* = .01),^16^ whereas its predictive value was not validated in different molecular subtypes of BC. Our previous study specifically focusing TNBC patients found that vertical orientation in preoperative ultrasound was associated with worse outcomes in terms of RFS and BCSS.^17^ However, its prognostic role in other molecular subtypes was still unknown. Thus, to further explore the prognostic value of tumor orientation in BC and its association with molecular subtypes, we conducted this study in a larger cohort of 2812 BC patients. It was demonstrated that the prognostic effect of tumor orientation was significantly associated with molecular subtypes (TNBC vs non‐TNBC) in predicting RFS (*P*_interaction_ = 0.001) and BCSS (*P_interaction_* = 0.001). Vertical orientation in TNBC was independently associated with worse RFS and BCSS, which was consistent with our previous study.^21^ Although similar results were found in ER‐negative BC, most RFS (14/17), and BCSS (12/14) events in vertical subgroup were among TNBC patients (Table 2), indicating the unfavorable prognostic value of vertical orientation in ER‐negative BC was mostly due to the TNBC cohort. Interestingly, for non‐TNBC patients, vertical orientation was associated with favorable RFS (*P* = .035) in univariate analysis, whereas had no predictive value in multivariate analysis. Furthermore, vertical orientation did not show significant prognostic values for RFS and BCSS (All *P* > .05) in Luminal‐A like, Luminal‐B like, and HER2‐enriched subtypes. However, when we looked at the 5‐year RFS in each subtype, tumors with vertical orientation had a tendency of better RFS compared with parallel orientation in Luminal‐B like (95.2% vs 92.1%) and HER2‐enriched (92.9% vs 88.8%) subgroups. The nonsignificant *P* values may be caused by relatively few recurrence events in each subgroup.

Vertical orientation, which meant the lesion was oriented taller than wide according to ACR Reporting system, was able to reflect the pathological features of BC.^23^ It was normally interpreted as high proliferation which led to expansive growth against peripheral tissues^24^ and thus was always considered as a risk sign for BC.^25^, ^26^ Meanwhile, studies have also manifested that vertical orientation was significantly associated with more invasive proportion of tumor^27^ and higher level of clinical risk in BC.^28^ However, evidence concerning vertical orientation and clinical outcomes in BC was inconsistent.^29^ Chae et al investigated a 267 cohort of ER+/HER2‐invasive BC and concluded that parallel orientation was an independent predictor for higher Oncotype DX RS (OR = 5.53; *P* = .02).^29^ Consistent with this conclusion, parallel orientation was also associated with vicious tumorous behavior in univariate analysis in our non‐TNBC cohort. What's more, vertical orientation had strong relevance with smaller tumor size, HER‐2 negativity, and lower Ki‐67 level among non‐TNBC patients in our study, which indicated less proliferative behaviors and potentially favorable prognosis. Similarly, several studies have found out that tumors with higher proliferation level may present as regular shapes, parallel orientations, and circumscribed margins. It was hypothesized that those lesions mimicked the morphology of benign lesions because of their rapid cellular proliferation.^30^, ^31^, ^32^

One of the most interesting findings of our study was the association between sonographic orientation and molecular subtypes in predicting BC outcomes. This may be explained by diverse imaging patterns among different molecular subtypes of BC. As shown in previous studies, TNBC had distinguishing ultrasound morphology including circumscribed margins, regular shapes, and posterior acoustic enhancement patterns compared with non‐TNBC tumors because of its distinct biological behavior.^33^, ^34^, ^35^ Furthermore, studies have found that radiomic phenotypes were associated with genomic pathways and protein expressions which contributed to tumor development.^36^, ^37^ Thus, certain sonographic features may reflect distinctive biological profiles in different subgroups of BC. In our study, vertical orientation represented favorable features including smaller size and lower level of Ki‐67 in non‐TNBC while was associated with a higher ALN burden among TNBC patients. Radio‐genomic research may be warranted to further understand the clinical implications of sonographic orientation in different molecular subtypes of BC.

Regarding inclusion criteria, patients who underwent neoadjuvant therapy were excluded from our study population. The reasons were that neoadjuvant therapy could bring alteration of pathological profiles to BC, including hormone receptor status, HER‐2 status, and Ki‐67 index, which caused the change in molecular subtypes and would further influence patients' long‐term prognosis.^38^, ^39^, ^40^ Additionally, neoadjuvant treatment would bring change to the sonographic morphology of breast tumors, which would cause bias to the preoperative imaging features. Thus, prognostic values of sonographic features in neoadjuvant settings should be analyzed separately from adjuvant settings.

There were certainly several limitations in this study. First, it was retrospectively designed and conducted in single institution, which may lead to selective and treatment bias among the study population. Second, 213 (7.6%) patients with IHC HER2 2+ lacked further FISH tests, causing a relatively large proportion of undetermined HER2 status and group of patients with unclassified molecular subtype in our population. Additionally, applications of novel features including quantitative ultrasound^41^ and shear wave elastography^42^ were unavailable in this study, which may better predict treatment response and survival in BC.

## 5 CONCLUSIONS

In conclusion, our study demonstrated that the prognostic value of sonographic orientation in predicting disease outcomes was associated with molecular subtypes. Vertical orientation was independently associated with inferior prognosis in TNBC. Hence, vertical orientation could be recognized as a candidate prognostic risk factor for TNBC, deserving further clinical evaluation.

## CONFLICT OF INTEREST

The authors of this manuscript declare no relationships with any companies, whose products or services may be related to the subject matter of the article.

## AUTHOR CONTRIBUTIONS

HY Wang was in charge of data analyses and manuscript writing. JJ Yao, Y Zhu, and WW Zhan made great contributions to imaging collecting and evaluation. K Shen and X Chen made equal contributions to the work of study design and manuscript revision.

## Supporting information

Supplementary Material

## Data Availability

The data that support the findings of this study are available from the corresponding author upon reasonable request.
