# Supplementary material for: Association of sonographic features and molecular subtypes in predicting breast cancer disease outcomes
Source: Cancer Med. 2020 Jul 13;9(17):6173–85. doi: 10.1002/cam4.3305 (PMC7476839; doi:10.1002/cam4.3305)
Supplement: Supplementary file 1 — Supplementary Material [file CAM4-9-6173-s001.docx]

**Supplementary Tables**

**Table S1:** Univariate analysis of sonographic features and clinical outcomes in patients with ER-positive and ER-negative BC.

| **Variables** | **ER-positive** | |  | **ER-negative** | |
| --- | --- | --- | --- | --- | --- |
|  | **RFS** | **BCSS** |  | **RFS** | **BCSS** |
| **Orientation (vertical vs parallel)** | 0.312 | 0.981 |  | **<0.001*** | **<0.001*** |
| **Shape (irregular vs regular)** | **0.022*** | 0.142 |  | 0.556 | 0.556 |
| **Margin** | 0.242 | 0.589 |  | 0.799 | 0.799 |
| Angular vs circumscribed | 0.062 | 0.172 |  | 0.362 | 0.362 |
| Spiculate vs circumscribed | 0.252 | 0.603 |  | 0.349 | 0.349 |
| Micro-lobulated vs circumscribed | 0.058 | 0.235 |  | 0.519 | 0.519 |
| Distinct vs circumscribed | 0.485 | 0.551 |  | 0.850 | 0.850 |
| **Posterior acoustic pattern** | 0.155 | 0.891 |  | 0.692 | 0.692 |
| Shadowing vs no change | 0.251 | 0.683 |  | 0.403 | 0.403 |
| Enhancement vs no change | 0.089 | 0.624 |  | 0.654 | 0.654 |
| Mixed change vs no change | 0.080 | 0.634 |  | 0.730 | 0.730 |
| **Calcification (yes vs no)** | 0.559 | 0.860 |  | 0.384 | 0.384 |
| **Architectural distortion (yes vs no)** | 0.188 | 0.568 |  | 0.505 | 0.505 |
| **Change in Cooper’s ligament (yes vs no)** | 0.224 | 0.491 |  | 0.525 | 0.525 |
| **CDFI** | 0.845 | 0.246 |  | 0.750 | 0.750 |
| Low vs no | 0.808 | 0.688 |  | 0.449 | 0.449 |
| High vs no | 0.648 | 0.312 |  | 0.517 | 0.517 |
| **BI-RADS (4B, 4C, 5 vs 4A)** | 0.559 | 0.391 |  | 0.374 | 0.358 |
| **BI-RADS (5 vs 4A, 4B, 4C)** | **0.041*** | **0.002*** |  | **0.001*** | **0.002*** |

**Abbreviations:** RFS, recurrence-free survival; BCSS, breast cancer-specific survival; ER, estrogen receptor; CDFI, color Doppler flow imaging; BI-RADS, Breast imaging reporting and data system.

**Table S2: Multivariate analysis of sonographic and clinicopathological features for RFS and BCSS in TNBC patients.**

| **Categories** | **RFS** |  | **BCSS** |  |
| --- | --- | --- | --- | --- |
|  | **SHR (95% CI)** | ***P* value** | **SHR (95% CI)** | ***P* value** |
| **Orientation** |  | **0.004** |  | **<0.001** |
| Parallel | 1.00 |  | 1.00 |  |
| Vertical | 3.10(1.43-6.68) |  | 6.41(2.94-13.99) |  |
| **Tumor size** |  | **0.001** |  | **0.016** |
| ≤2cm | 1.00 |  | 1.00 |  |
| >2cm | 2.79(1.50-5.18) |  | 2.63(1.18-5.91) |  |
| **ALN metastases** |  | 0.027 |  | **0.007** |
| No | 1.00 |  | 1.00 |  |
| Yes | 2.05(1.08-3.89) |  | 3.12(1.36-7.15) |  |
| **LVI** |  | **0.004** |  | **0.005** |
| No | 1.00 |  | 1.00 |  |
| Yes | 3.19(1.46-7.01) |  | 3.76(1.50-9.46) |  |
| **Histological grade** |  | **0.048** |  | **0.210** |
| I-II | 1.00 |  | 1.00 |  |
| III | 2.25(1.01-5.03) |  | 1.82(0.71-4.66) |  |
| **Chemotherapy** |  | **0.013** |  | **0.032** |
| No | 1.00 |  | 1.00 |  |
| Yes | 0.32(0.13-0.79) |  | 0.34(0.13-0.90) |  |

**Abbreviation:** RFS, recurrence-free survival; BCSS, breast cancer-specific survival; TNBC, triple negative breast cancer; ALN, axillary lymph node; LVI, lymphatic vessel invasion; SHR, sub-distribution hazard ratio; CI, confidence interval.

**Table S3:** Univariate analysis of orientation and patient outcomes in different molecular subtypes of BC patients

| **Endpoints** | **Vertical** | |  | **Parallel** | | ***P* value** |
| --- | --- | --- | --- | --- | --- | --- |
|  | **Events**  **N** | **5Yr rate**  **%** |  | **Events**  **N** | **5Yr rate**  **%** |  |
| **RFS** |  |  |  |  |  |  |
| Non-TNBC | 17 | 96.0 |  | 166 | 92.9 | **0.035*** |
| Luminal A-like | 3 | 98.4 |  | 15 | 98.3 | 0.713 |
| Luminal B-like | 12 | 95.2 |  | 117 | 92.1 | 0.095 |
| HER2-enriched | 2 | 92.9 |  | 34 | 88.8 | 0.531 |
| **BCSS** |  |  |  |  |  |  |
| Non-TNBC | 6 | 98.4 |  | 64 | 96.8 | 0.207 |
| Luminal A-like | 0 | 100.0 |  | 6 | 96.8 | 0.236 |
| Luminal B-like | 5 | 98.1 |  | 46 | 96.5 | 0.351 |
| HER2-enriched | 1 | 93.7 |  | 12 | 95.1 | 0.734 |

**Abbreviation:** TNBC, triple negative breast cancer; ER, estrogen receptor; RFS, recurrence-free survival; BCSS, breast cancer-specific survival.

**Table S4: Differences of clinicopathological features between vertical and parallel orientation in TNBC patients.**

|  | **TNBC** | | |
| --- | --- | --- | --- |
| **Variables** | **Vertical (N=48)** | **Parallel (N=368)** | ***P* value** |
| **Age (y)** |  |  | **0.138** |
| <35 | 0(0.0) | 19(5.2) |  |
| 35-44 | 6(12.5) | 55(14.9) |  |
| 45-54 | 12(25.0) | 116(31.5) |  |
| 55-64 | 19(39.6) | 90(24.5) |  |
| ≥65 | 11(22.9) | 88(23.9) |  |
| **Menstrual status** |  |  | **0.218** |
| Pre/peri-menopausal | 15(31.3) | 149(40.5) |  |
| Postmenopausal | 33(68.8) | 219(59.5) |  |
| **Surgery time interval** |  |  | **0.116** |
| ≤6 days | 27(56.3) | 249(67.7) |  |
| >6 days | 21(43.7) | 119(32.3) |  |
| **Histopathological type** |  |  | **0.792** |
| IDC | 44(91.7) | 333(90.5) |  |
| Others | 4(8.3) | 35(9.5) |  |
| **LVI** |  |  | **0.568** |
| Absent | 46(95.8) | 345(93.8) |  |
| Present | 2(4.2) | 23(6.3) |  |
| **Histological grade** |  |  | **0.066** |
| I | 0(0.0) | 1(0.3) |  |
| II | 19(39.6) | 94(25.5) |  |
| III | 22(45.8) | 233(63.3) |  |
| NA | 7(14.6) | 40(10.9) |  |
| **Tumor size** |  |  | **0.351** |
| ≤2cm | 26(54.2) | 173(47.0) |  |
| >2cm | 22(45.8) | 195(53.0) |  |
| **Lymph nodes involvement** |  |  | **0.001*** |
| mean±SE | 2.8±1.0 | 1.4±0.2 |  |
| **Ki-67%** |  |  | **0.039*** |
| mean±SE | 43.6±4.4 | 53.4±1.4 |  |

**Abbreviations:** TNBC, triple-negative breast cancer; IDC, invasive ductal carcinoma; LVI, lymphovascular invasion; SE, standard error.

**Table S5:** Differences of clinicopathological features between vertical and parallel orientation in ER-negative and ER-positive patients.

| **Variables** | **ER-positive** | | |  | **ER-negative** | | |
| --- | --- | --- | --- | --- | --- | --- | --- |
|  | **Vertical (N=291)** | **Parallel (N=1755)** | **P value** |  | **Vertical (N=72)** | **Parallel (N=694)** | **P value** |
| **Age (yrs)** |  |  | **<0.001** |  |  |  | **0.161** |
| ≤55 | 115(39.5) | 896(51.1) |  |  | 31(43.1) | 359(51.7) |  |
| >55 | 176(60.5) | 859(48.9) |  |  | 41(56.9) | 335(48.3) |  |
| **Menstrual status** |  |  | **0.001** |  |  |  | **0.124** |
| Pre/peri-menopausal | 91(31.3) | 729(41.5) |  |  | 19(26.4) | 246(35.4) |  |
| Postmenopausal | 200(68.7) | 1026(58.5) |  |  | 53(73.6) | 448(64.6) |  |
| **Co-morbidity** |  |  | **<0.001** |  |  |  | **0.475** |
| Absent | 147(50.5) | 1079(61.5) |  |  | 48(66.7) | 433(62.4) |  |
| Present | 144(49.5) | 676(38.5) |  |  | 24(33.3) | 261(37.6) |  |
| **Histopathological type** |  |  | **0.492** |  |  |  | **0.293** |
| IDC | 259(89.0) | 1537(87.6) |  |  | 67(93.1) | 618(89.0) |  |
| Others | 32(11.0) | 218(12.4) |  |  | 5(6.9) | 76(11.0) |  |
| **LVI** |  |  | **0.054** |  |  |  | **0.759** |
| Absent | 284(97.6) | 1668(95.0) |  |  | 68(94.4) | 649(93.5) |  |
| Present | 7(2.4) | 87(5.0) |  |  | 4(5.6) | 45(6.5) |  |
| **Tumor grade** |  |  | **0.155** |  |  |  | **0.246** |
| I-II | 185(69.5) | 1002(65.1) |  |  | 21(32.8) | 163(26.1) |  |
| III | 81(30.5) | 538(34.9) |  |  | 43(67.2) | 462(73.9) |  |
| **Tumor size** |  |  | **<0.001** |  |  |  | **0.070** |
| ≤2cm | 224(77.0) | 992(56.5) |  |  | 40(55.6) | 308(44.4) |  |
| >2cm | 67(23.0) | 763(43.5) |  |  | 32(44.4) | 386(55.6) |  |
| **HER2** |  |  | **0.007** |  |  |  | **0.005** |
| Negative | 226(89.7) | 1343(82.3) |  |  | 51(75.0) | 376(57.2) |  |
| Positive | 24(9.5) | 281(17.2) |  |  | 17(25.0) | 281(42.8) |  |
| Undetermined | 2(0.8) | 8(0.5) |  |  | 0(0.0) | 0(0.0) |  |
| **Lymph nodes involvement** |  |  | **0.205** |  |  |  | **0.049*** |
| mean±SE | 1.3±0.9 | 1.6±0.1 |  |  | 2.9±0.2 | 1.7±0.1 |  |
| **Ki-67%** |  |  | **0.001** |  |  |  | **0.078** |
| mean±SE | 18.6±1.1 | 22.5±0.5 |  |  | 41.0±3.3 | 47.0±1.0 |  |

**Abbreviations:** IDC, invasive ductal carcinoma; LVI, lymphovascular invasion; ER, estrogen receptor; PR, progesterone receptor; HER2, human epidermal growth factor receptor 2; SE, standard error; HR, hazard ratio; CI, confidence interval.

**Supplementary Figures**

**Figure S1:** Kaplan-Meier survival curves for sonographic orientation in ER-negative and ER-positive patients.


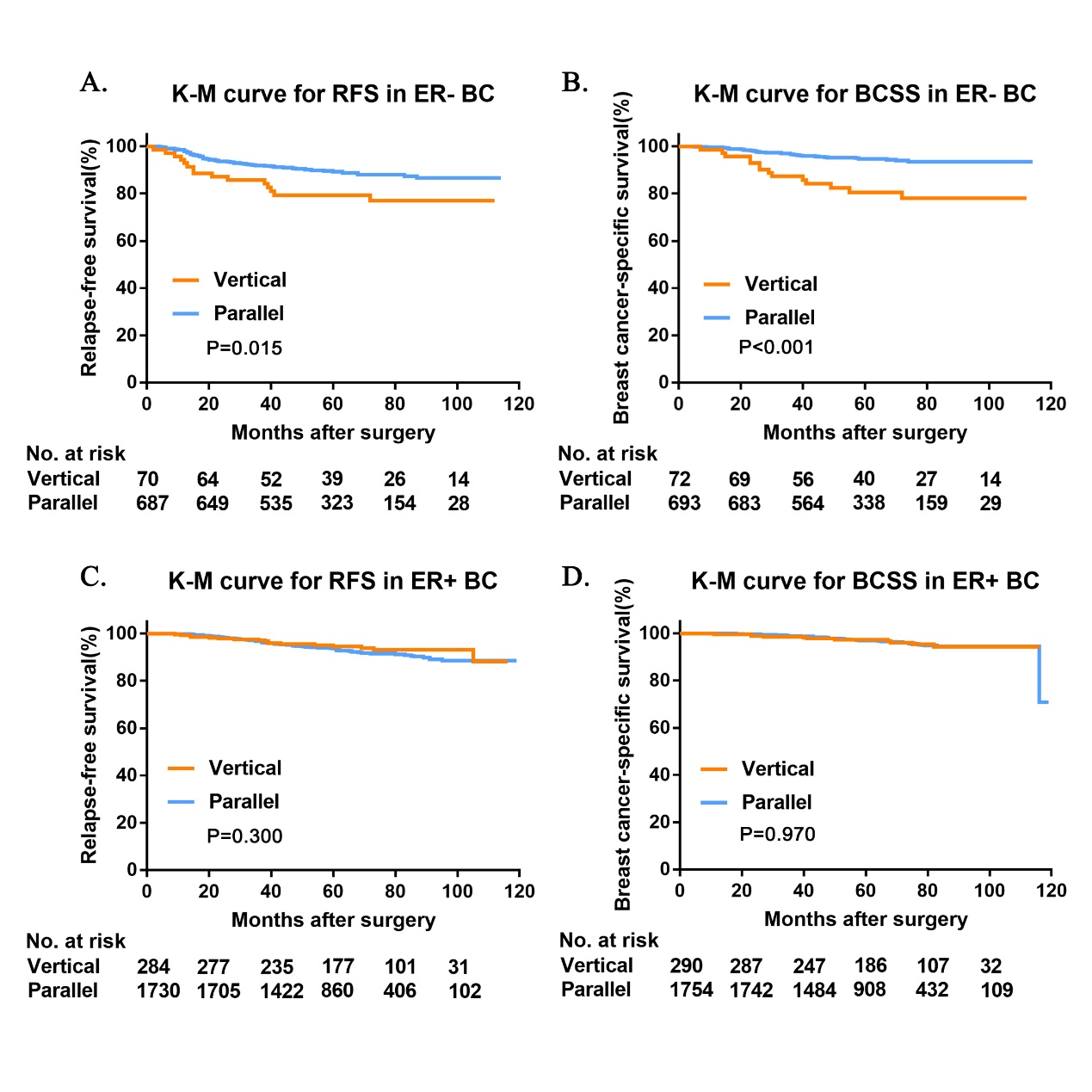


Figure legend: Kaplan-Meier curves stratified by sonographic orientations were illustrated for estrogen receptor (ER)-negative BC and ER-positive BC patients respectively. Vertical orientation predicted superior A) RFS (P=0.015) and B) BCSS (P<0.001) for ER-negative BC while showed similar C) RFS (P=0.300) and D) BCSS (P=0.970) compared with parallel tumors for ER-positive BC.

Abbreviations: ER, estrogen receptor; BC, breast cancer; K-M, Kaplan-Meier; RFS, recurrence-free survival; BCSS, breast cancer-specific survival.

**Figure S2:** Kaplan-Meier survival curves for sonographic orientation in Luminal-A like, Luminal B-like and HER2-enriched BC patients.


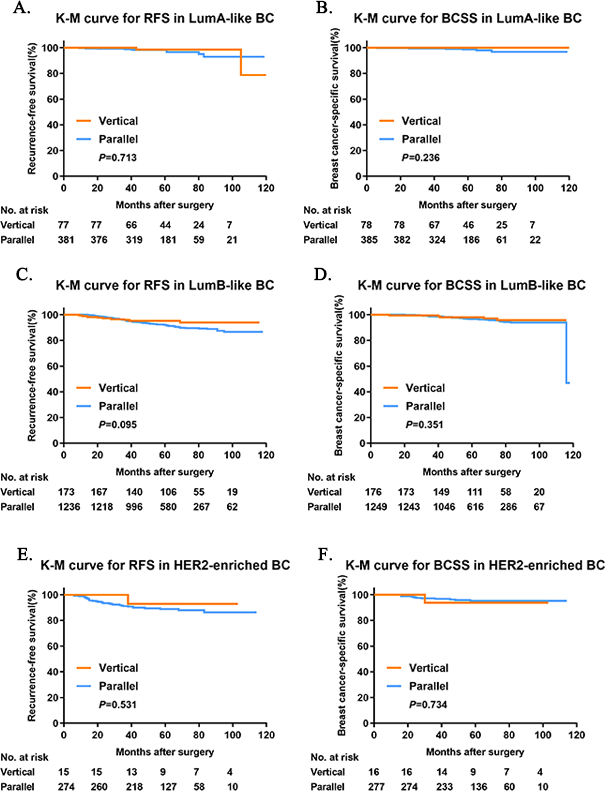


Figure legend: Kaplan-Meier curves for RFS and BCSS stratified by sonographic orientations were plotted for Luminal-A like, Luminal-B like and HER2-enriched BC patients separately. Vertical orientation failed to predicted A) RFS (*P*=0.713) and B) BCSS (*P*=0.236) in Luminal-A like BC; C) RFS (*P*=0.095) and D) BCSS (*P*=0.351) in Luminal-B like BC; and E) RFS (*P*=0.531) and F) BCSS (*P*=0.734) in HER2-enriched BC patients.

Abbreviations: BC, breast cancer; HER2, human epidermal growth factor receptor 2; K-M, Kaplan-Meier; RFS, recurrence-free survival; BCSS, breast cancer-specific survival.
